# Case Report: A Rosette-forming Glioneuronal Tumor in the Tectal Plate in a Patient with Neurofibromatosis Type I

**DOI:** 10.7759/cureus.857

**Published:** 2016-11-01

**Authors:** Emily P Sieg, Russell Payne, Sara Langan, Charles S Specht

**Affiliations:** 1 Department of Neurosurgery, Penn State Hershey Medical Center; 2 Pathology, Penn State Hershey Medical Center

**Keywords:** neurofibromatosis type 1 (nf1), rosette-forming glioneuronal tumor (rgnt), obstructive hydrocephalus

## Abstract

We report the case of a 41–year-old female with neurofibromatosis Type 1 (NF1) who developed a rosette-forming glioneuronal tumor (RGNT) in the tectal plate. This tumor was diagnosed in 2002 when the patient presented with obstructive hydrocephalus, which was subsequently treated with a ventriculoperitoneal shunt and then an endoscopic third ventriculostomy. Initially thought to be a pilocytic astrocytoma, it was followed with serial magnetic resonance imaging (MRI) until tumor progression and development of a large fourth ventricular cystic component prompted resection via suboccipital craniotomy. Histological examination demonstrated an RGNT, a WHO Grade 1 tumor, with neurocytic rosettes, perivascular pseudorosettes, and elements resembling a pilocytic astrocytoma. Initially, the patient did well after her craniotomy, but postoperative complications set in that eventually led to her death. In this case report, we describe a relatively rare tumor that, despite its benign nature, leads to frequent complications and deficits due to its surgically challenging location. Along with previously reported examples, this cases raises the possibility of a causal relationship between NF1 and RGNT.

## Introduction

Rosette-forming glioneuronal tumor (RGNT) is a rare neoplasm. This lesion was first described as a distinct entity with a series of 11 cases by Komori, et al. in 2002 [1]. This neoplasm is found in the WHO 2007 Classification of Tumours of the Central Nervous System within the group of “neuronal and mixed glioneuronal tumors”, a category that includes neurocytoma, ganglioglioma/gangliocytoma, dysembryoplastic neuroepithelial tumor, and similar lesions [2].

The radiologic and histopathologic features of RGNT are distinctive [1-3]. This slow growing tumor occurs predominantly in the midline, where it involves the brainstem and cerebellum. It affects young to middle-aged adults (mean age: 33) with a female predominance of 1.9 to 1. Given their location, most RGNTs present with symptoms of obstructive hydrocephalus, headache, and ataxia. RGNT is a WHO Grade I tumor and is curable with complete resection. These tumors are associated biologically with a favorable prognosis, but postoperative complications and deficits due to the location of the tumor are frequent. RGNT was initially described as a fourth ventricular lesion, but similar lesions have been reported in the brainstem, cerebellum, optic chiasm, spinal cord, pineal region, and lateral and third ventricle.

A possible association of RGNT with neurofibromatosis Type 1 (NF1) has been suggested [4-6]. Here, we report a patient with NF-1 and an RGNT in the tectal region that extended with a cystic component into the fourth ventricle. 

## Case presentation

Informed patient consent was obtained for treatment. No identifying patient information is contained in this report.

**Figure 1:**
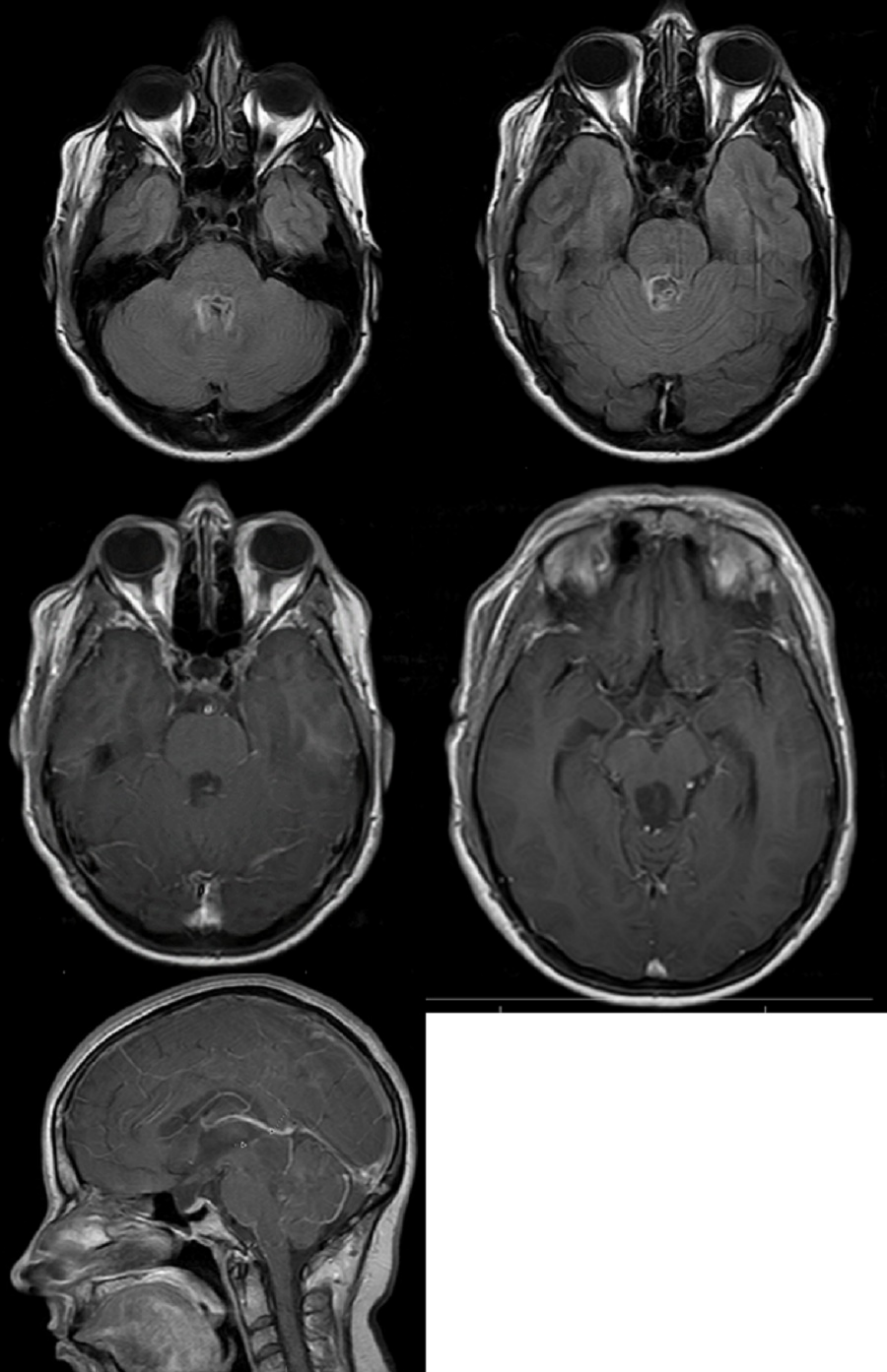
T1 Axial Ill-defined mass originating from the tectal/pineal region and extending inferiorly and occupying the fourth ventricle. Hypointense on T1 images.

The patient was a 41-year-old right-handed female who had been diagnosed with NF1 at age 6. With the exception of multiple cutaneous manifestations of NF1, she did well until 2002 when signs, symptoms, and radiographic evidence led to the diagnosis of a large tectal lesion with compression of the brainstem and obstructive hydrocephalus. This was treated with a ventriculoperitoneal (VP) shunt. She did well until 2007 when her headaches and gait imbalance recurred. Ultimately, the VP shunt was removed and an endoscopic third ventriculostomy was performed. She continued to have headaches and dizziness. The tumor had been followed with serial MRIs. The lesion was stable until May 2012, when enlargement of a cystic component with compression of the brainstem was documented on MRI (Figure 1).

**Figure 2:**
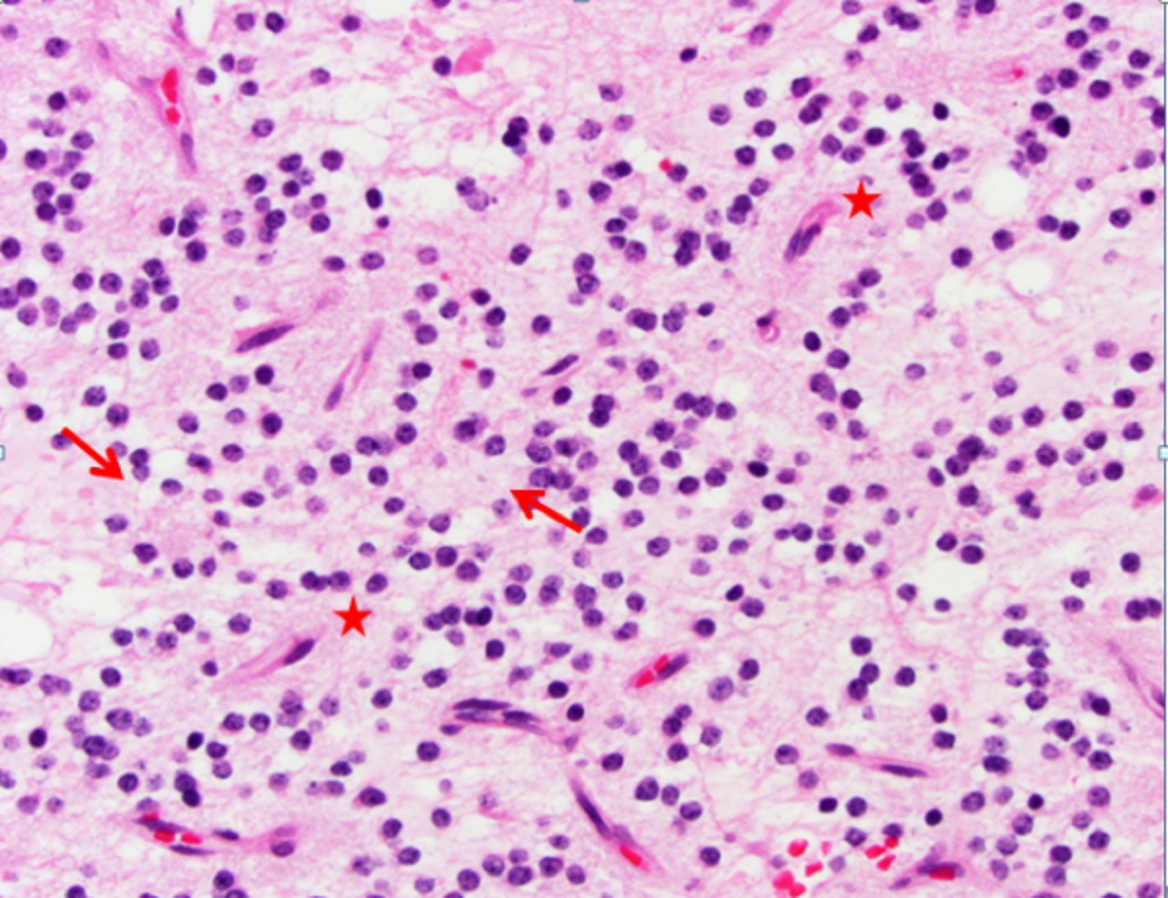
H&E, 500x Rosette-forming glioneuronal tumor (RGNT) neurocytic cells with round nuclei. These cells form rosettes (arrows) and perivascular pseudorosettes (stars).

**Figure 3:**
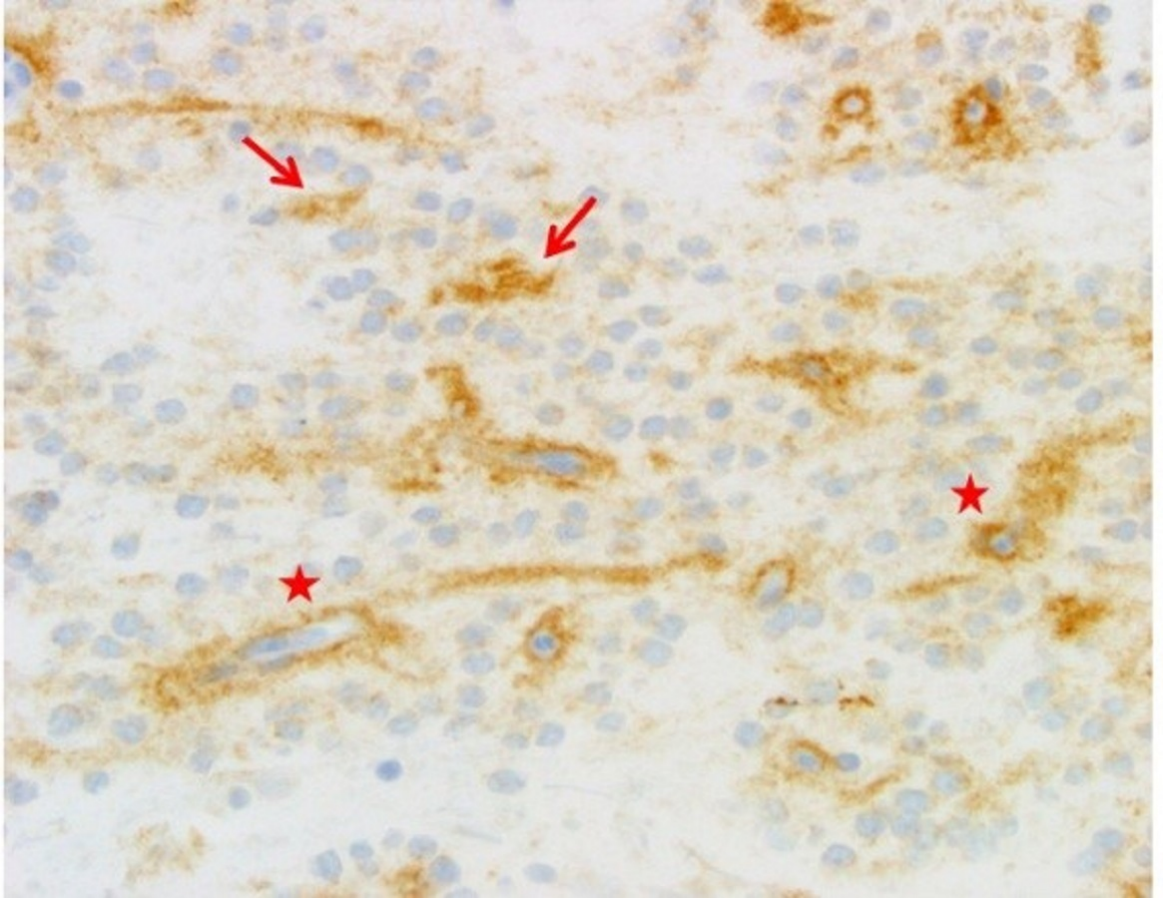
Synaptophysin, 500x Note the pattern of brown synaptophysin staining around capillaries in the neurocytic perivascular pseudorosettes (stars) and in the centers of the true neurocytic rosettes (arrows).

The patient underwent suboccipital transvermian craniotomy for subtotal resection of the tumor in October 2012. Pathology showed a low cellularity neoplasm with two major components (Figures 2-3). These included glial fibrillary acidic protein (GFAP)-positive areas of piloid astrocytic cells resembling pilocytic astrocytoma and other areas with synaptophysin-positive neurocytic tumor cells that formed neurocytic rosettes or perivascular pseudorosettes. Dysmorphic ganglion cells were focally noted as was focal myxoid change. Rosenthal fibers and Periodic acid–Schiff–diastase (PASD)-positive eosinophilic granular bodies (EGBs) were seen. Mitotic figures were not identified. The Ki-67 nuclear labeling index was low at about 2% in the most proliferative areas.  

The initial postoperative course was uneventful, and the patient was discharged for rehabilitation. However, about 2 ½ weeks postoperatively, she developed a pseudomeningocele. This was followed by pseudomonas meningitis and recurrent hydrocephalus. Over the next seven months, she had a very complicated course with multiple episodes of meningitis, urinary sepsis, and Clostridium difficile colitis. After a slow but progressive decline in functional status and multiple hospitalizations for complicated infections, her family made the decision for patient comfort care only. She expired in home hospice. 

## Discussion

Rosette-forming glioneuronal tumor (RGNT) is a rare tumor that was initially described as a tumor of the fourth ventricle [1-2]. This lesion has since been documented in other midline locations within the cerebellum, brainstem, and spinal cord. Fourth ventricular examples of RGNT typically present, as seen in our patient, with ataxia or headaches due to obstructive hydrocephalus [1, 3]. RGNT has a highly distinctive histological appearance with neurocytic rosettes/perivascular pseudorosettes and elements that resemble a pilocytic astrocytoma. It was included as a Grade 1 tumor entity in the WHO 2007 Classification of Tumours of the Central Nervous System [2]. 

This case elucidates the need for possible changes in surgical techniques for these tumors. Other benign tumors in eloquent areas can be treated with stereotactic means, e.g. Gamma Knife, stereotactic laser-induced thermotherapy (LITT), before they progress and become occlusive or a surgical emergency; should this be considered for RGNT? Also, is it worth biopsying these tumors? All/nearly all brain tumors associated with NF1 are Grade 1, often in eloquent areas. Perhaps new surgical techniques/thinking needs to be applied to avoid surgical morbidity in benign lesions? 

Three case reports have been published documenting patients with NF1 and RGNT [4-6]. Our patient had neurofibromatosis Type 1 (NF1) and so represents a fourth example of this association. Thus, the possibility of a causal relationship between NF1 and RGNT should be considered. An intriguing recent study indicates that missense mutations of PIK3CA are a common event in RGNT [7]. PIK3CA is the primary catalytic component of PI3K and, thus, drives the PI3K/AKT/mTOR pathway, a principal regulator of cell metabolism [8]. Missense mutations often lead to the gain of PIK3CA function [8]. A gain-of-function mutation in the PI3K family would lead to increased activity of the PI3K/AKT/mTOR pathway. Both the PI3K/AKT/mTOR and RAF/MEK/ERK pathways are driven by the RAS oncogene [9]. These pathways control many cellular functions associated with growth, proliferation, and apoptosis and, thus, are principal players in the development of many neoplasms [8-9]. In patients with NF1, the normal regulation of RAS is lost and both of these pathways show increased activity with associated tumorigenesis [9]. NF1 and its associated central nervous system tumors have been well-described in the literature and include brain and spinal cord gliomas, optic pathway gliomas, and dysembryoplastic neuroepithelial tumors (10).

## Conclusions

NF1 and its associated central nervous system tumors have been well-described in the literature and include brain and spinal cord gliomas, optic pathway gliomas, and dysembryoplastic neuroepithelial tumors [10].^ ^As mentioned previously, there are an increasing number of cases that have reported RGNT development in the setting of NF1, and molecular studies have implicated similar mechanisms of tumorigenesis. Therefore, a causal relationship between the loss of RAS oncogene regulation leading to increased P13K/AKT/mTOR pathway activity and subsequent development of RGNT in patients with NF1 should be considered.  
